# Intravitreal Dexamethasone Implant for Postoperative Macular Oedema Secondary to Vitrectomy for Epiretinal Membrane and Retinal Detachment: A Systematic Review and Meta-Analysis

**DOI:** 10.1155/2021/6627677

**Published:** 2021-04-16

**Authors:** Guglielmo Parisi, Matteo Fallico, Teresio Avitabile, Antonio Longo, Elina Ortisi, Andrea Russo, Francesco Petrillo, Andrea Maugeri, Martina Barchitta, Vincenza Bonfiglio, Claudio Furino, Gilda Cennamo, Paolo Caselgrandi, Paola Marolo, Luca Ventre, Michele Reibaldi

**Affiliations:** ^1^Department of Surgical Sciences, Eye Clinic Section, University of Turin, Turin 10122, Italy; ^2^Department of Ophthalmology, University of Catania, Catania 95100, Italy; ^3^Department of Medical and Surgical Sciences and Advanced Technologies “GF Ingrassia”, University of Catania, Catania, Italy; ^4^Department of Experimental Biomedicine and Clinical Neuroscience, Ophthalmology Section, University of Palermo, Palermo 90127, Italy; ^5^Department of Ophthalmology, University of Bari, Bari, Italy; ^6^Department of Public Health, University of Naples Federico II, Naples, Italy

## Abstract

**Purpose:**

To evaluate the efficacy of intravitreal dexamethasone implant (DEX) for the treatment of macular oedema secondary to vitrectomy for epiretinal membrane (ERM) and retinal detachment (RD) by conducting a systematic review with meta-analysis of published studies.

**Methods:**

Studies reporting clinical outcomes of DEX use for the treatment of macular oedema secondary to ERM and RD vitrectomy were searched on PubMed and Embase databases. The primary outcome was best-corrected visual acuity (BCVA) change between baseline and post-DEX treatment, reported as mean difference (MD) with 95% confidence interval (CI). Mean central macular thickness (CMT) change was assessed as a secondary outcome. Postimplant adverse events, including intraocular pressure rise and cataract development, were reported as well.

**Results:**

Five uncontrolled studies, 1 nonrandomized controlled study, and 1 randomized controlled study were included, with a total of 5 cohorts and 3 cohorts in the ERM group and RD group, respectively. Considering the last available follow-up, a significant improvement in postimplant BCVA was found in the overall population, irrespective of the indication for vitrectomy (MD = −0.28, 95% CI = −0.37, −0.20; *p* < 0.001), but with significant heterogeneity. In either group, mean BCVA significantly improved following the implant (in the ERM group, MD = −0.31, 95% CI = −0.40, −0.22; in the RD group, MD = −0.22, 95% CI = −0.41, −0.03), with no difference between the two groups (*p*=0.41). However, there was significant heterogeneity in both groups. Considering the last available follow-up, a significant CMT reduction was found in the overall population, irrespective of the indication for vitrectomy (MD = −129.75, 95% CI = −157.49, −102.01; *p* < 0.001). In the ERM group, a significant CMT reduction was shown following DEX (MD = −133.41, 95% CI = −155.37, −111.45; *p* < 0.001), with no heterogeneity. In the RD group, mean CMT reduction was borderline significant (MD = −128.37, 95% CI = −253.57, −3.18; *p*=0.040), with significant heterogeneity. No difference in CMT improvement was found between the two groups (*p*=0.94).

**Conclusion:**

This meta-analysis showed that DEX yielded a significant improvement in visual and anatomical outcomes, even if limited by significant heterogeneity. Dexamethasone implant represents an effective treatment for postoperative macular oedema secondary to ERM and RD vitrectomy.

## 1. Introduction

Postoperative cystoid macular oedema (CMO) represents one of the main causes of postoperative visual impairment, generally occurring between 4 and 12 weeks after surgery [1]. This condition has been also reported following vitrectomy, with an incidence as high as 47% of cases [2]. Its etiology mainly depends on an inflammatory process triggered by the surgery [3, 4].

For such a reason, steroids have been widely used for the treatment of postoperative CME, including the sustained-release dexamethasone intravitreal implant (Ozurdex®, Allergan Inc., Irvine, CA, USA, and Allergan Pharmaceuticals, Ireland) [5].

In particular, several authors reported the use of intravitreal dexamethasone implant (DEX) for macular oedema secondary to vitrectomy for epiretinal membrane (ERM) and rhegmatogenous retinal detachment (RRD), showing promising results [6–13]. However, most of these studies were limited by a small sample size and retrospective design.

To date, no systematic review has been conducted with the purpose of analysing outcomes of DEX for the treatment of postvitrectomy CMO. Such a study would provide a clearer picture of both the potential benefits and drawbacks of this therapeutic option.

Therefore, we systematically reviewed the scientific evidence on the use of DEX for macular oedema secondary to vitrectomy for ERM and RRD and performed meta-analyses on visual and anatomical outcomes.

## 2. Materials and Methods

### 2.1. Literature Search

The methodology was based on the statements reported by the Preferred Reporting Items for Systematic Reviews and Meta-Analyses (PRISMA) [14] (Table S1 available online in the Supplementary Material) and the Cochrane Handbook [15].

Studies reporting clinical outcomes of intravitreal dexamethasone implant for the treatment of postoperative macular oedema after vitrectomy for ERM or RRD were systematically reviewed. An electronic search of PubMed and Embase databases was carried out. The search method included the terms “dexamethasone implant,” “vitrectomy,” “retinal detachment,” “epiretinal membrane,” “pucker,” and “macular oedema,” connected in various combinations by “and/or.” The last search was done on November 30, 2020. Studies published in peer-reviewed journals and in the English language were assessed for eligibility, regardless of publication date or status. If clarifications were needed, we contacted the authors by e-mail.

### 2.2. Eligibility Criteria

The following inclusion criteria were considered (1) to include patients with macular oedema secondary to vitrectomy for ERM and/or RRD, (2) to report clinical outcome of treatment with intravitreal dexamethasone implant, and (3) to present a follow-up ≥3 months.

The following exclusion criteria were adopted: (1) cohorts including patients receiving vitrectomy for diseases different from ERM or RRD; (2) cohorts receiving DEX for the prevention of macular oedema; and (3) a case report design.

The primary outcome measures were mean best-corrected visual acuity (BCVA) change and mean central macular thickness (CMT) change following dexamethasone implant administration.

### 2.3. Data Extraction and Quality Assessment

The eligibility of the studies was independently assessed by two investigators (G.P. and P.M.), who also carried out data extraction in an independent fashion. A third investigator (M.R.) was involved in case of disagreement. From each included article, the following data were extracted: year; location; first author; study design; number of patients; mean age; follow-up; indication for vitrectomy; type of surgery; time between surgery and macular oedema onset; time between surgery and DEX; type of treatment prior to dexamethasone; amount of intravitreal dexamethasone implant administered; and CMT-, BCVA-, DEX-related adverse events, including intraocular pressure (IOP) rise, cataract, infection. Extracted data on BCVA and CMT included pre-DEX, baseline values, and post-DEX values recorded throughout the follow-up of each study. In particular, post-DEX data included 1-month, 3-month, 6-month, and 12-month follow-up, if available.

The risk of bias of randomized studies was evaluated by using the Cochrane Handbook tool [15], while nonrandomized studies were assessed by using the methodological item for nonrandomized studies, as previously reported [16, 17].

### 2.4. Statistical Analysis

Best-corrected visual acuity was reported as logarithm of the minimum angle of resolution (logMAR). For both BCVA and CMT, the mean difference (MD) between baseline and post-DEX treatment values (i.e., last available follow-up and specific time-points such as 1-month, 3-month, 6-month, and 12-month follow-up) was calculated along with 95% confidence interval (95% CI). The *Q*-statistics and the *I*^2^ index were used to assess heterogeneity across studies. When significant heterogeneity was found (*I*^2^> 50% and Q-statistics <0.1), meta-analysis was based on a random effect approach, by applying the DerSimonian–Laird method. Otherwise, a fixed-effect model was used. Publication bias was evaluated by visual inspection of funnel plots along with Egger's test. Statistical analyses were conducted on STATA software (version 16). A *p* value <0.05 was considered significant for all analyses.

## 3. Results

### 3.1. Selection of Studies

**Figure 1:**
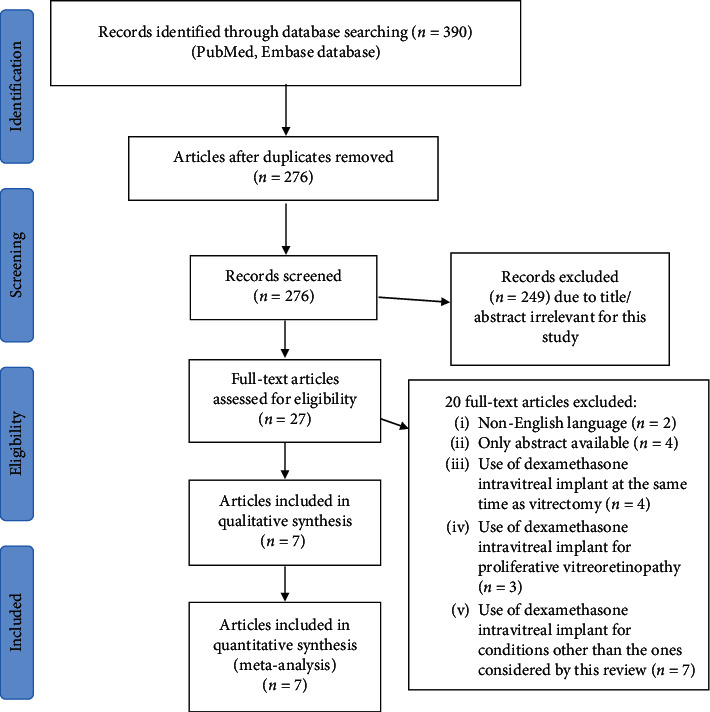
Flow diagram of the study selection process.

Figure 1 illustrates the study selection process. The electronic search allowed to identify a total of 390 articles, of which 114 were duplicates. Abstracts and titles of the remaining 276 articles were screened, and 27 potentially eligible studies were selected for full-text review. Of these, 20 studies were excluded. A total of 7 studies were included in this systematic review and were pooled together for meta-analyses.

### 3.2. Study Characteristics

Overall, 7 studies were included in this systematic review, of which 5 were uncontrolled retrospective reports [8–10, 12, 13]; one was a nonrandomized, retrospective, controlled study [11]; and one was a randomized controlled study [6]. All the included studies were published in years between 2014 and 2020. Overall, a total of 174 eyes were included, of which 46 eyes underwent vitrectomy for retinal detachment and 128 eyes for epiretinal membrane. The main characteristics of included studies are shown in Table 1. Freissinger et al. [9] reported outcomes of two cohorts: one including eyes with macular oedema secondary to ERM vitrectomy and the other including eyes with macular oedema secondary to RRD vitrectomy.

The nonrandomized retrospective controlled study included 40 eyes with long-term macular oedema after vitrectomy for ERM, of which 20 eyes received a single DEX implant and 20 eyes were untreated controls [11]. The results showed better BCVA and macular thickness in the DEX group compared to the control group. These improvements were maintained throughout the 6-month follow-up, even if macular thickness tended to increase at 6 months [11]. Only data from the DEX group were used for our pooled analyses.

The randomized controlled trial enrolled eyes diagnosed with macular oedema secondary to vitrectomy for ERM, which were randomized into two groups: a group receiving DEX implant (15 eyes) and a control, untreated group (12 eyes) [6]. Eyes treated with DEX had a significant improvement in both BCVA and macular thickness compared with the control group at 1-, 6-, and 12-month follow-up. A mean of 1.2 DEX injections was administered during the 12-month study period [6]. Only data from the DEX group were used for our pooled analyses.

No case of endophthalmitis was reported by included studies. The lens status of enrolled patients is shown in Table 1. With regard to IOP rise following DEX, this was recorded in 3 cases out of 39 by Hattenbach et al. [8], in 3 cases out of 20 by Chang et al. [11], in 3 cases out of 15 by Chatziralli et al. [6], in 11 cases out of 61 by Freissinger et al. [9], in 2 cases out of 14 by Chatziralli et al. [13], and in 3 cases out of 17 by Thanos et al. [10]. Furino et al. [12] reported no case of increased IOP. In all studies, IOP rise was successfully managed with IOP lowering drops, with no need for glaucoma surgery.

### 3.3. Quality Assessment

Table S2 (available online in Supplementary Material) illustrates the risk of bias of nonrandomized studies. The only randomized trial was judged at unclear risk for selection bias; performance bias and detection bias were deemed as an unclear risk; attrition bias and reporting bias were considered as low risk; and risk of other bias was unclear [6]. Funnel plots inspection and Egger's test showed no evidence of publication bias for the visual outcome (Figure S1 available online in Supplementary Material). Similarly, no evidence of publication bias was found for CMT change in the ERM group (Figure S2 available online in Supplementary Material). Egger's test revealed a risk of publication bias for CMT change in the retinal detachment group.

### 3.4. Visual Outcome

**Figure 2:**
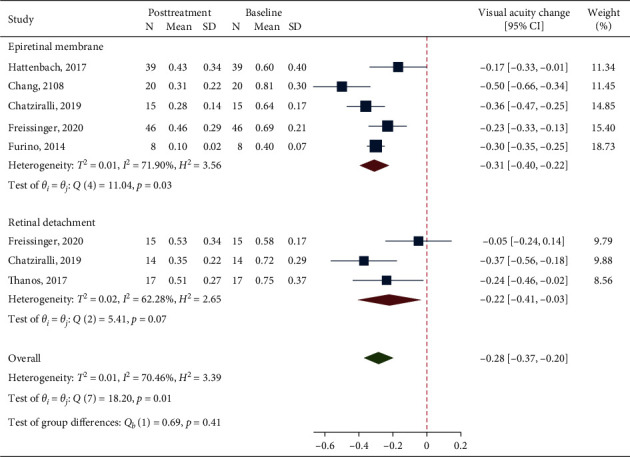
A forest plot showing the mean change in best-corrected visual acuity considering the last available follow-up after treatment with dexamethasone implant.

Data from 5 studies and 3 studies were pooled together for BCVA analysis in the ERM and RRD groups, respectively. The analysis on BCVA change between baseline and last available follow-up after DEX showed a significant visual improvement in the overall population, irrespective of the indication for vitrectomy (MD = −0.28, 95% CI = -0.37, −0.20; *p* < 0.001; Figure 2). However, significant heterogeneity was found (*I*^2^ = 70.5%; *p*=0.01). A MD of −0.31 (95% CI = −0.40, −0.22) was found in the ERM group and a MD of −0.22 (95% CI = −0.41, −0.03) was found in the RRD group, showing in both cases a significant BCVA improvement following dexamethasone implant (*p* values <0.001), with no difference between the two groups (*p*=0.41; Figure 1). However, heterogeneity was significantly high in both groups (*I*^2^ = 71.9% and *p*=0.03 for ERM; *I*^2^ = 62.3% and *p*=0.07 for RRD).

**Figure 3:**
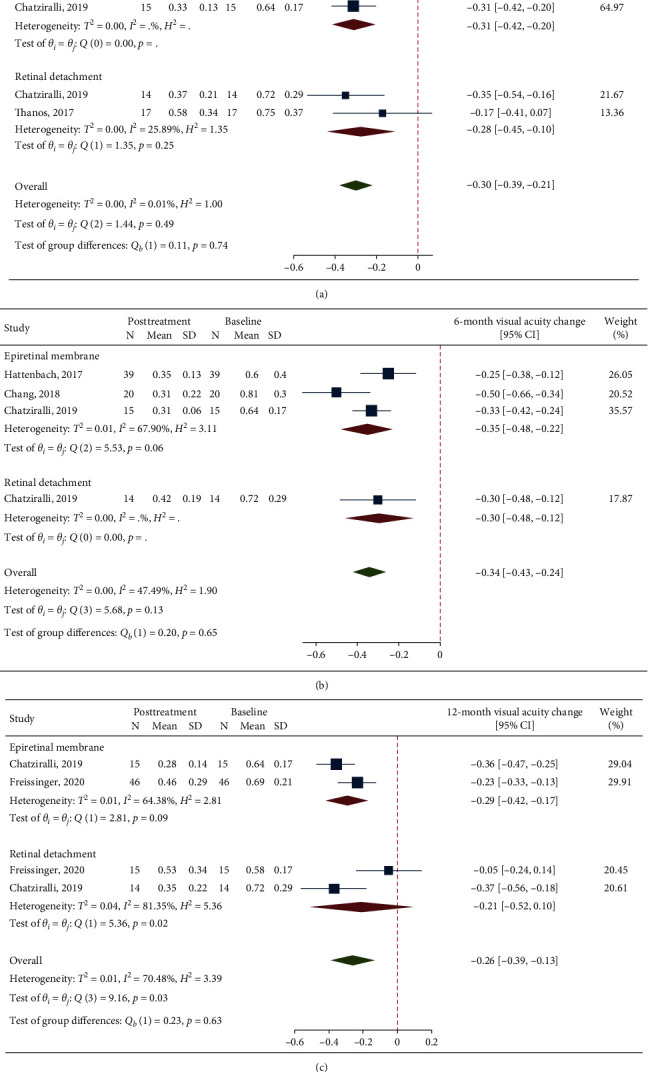
A forest plot showing mean change in best-corrected visual acuity at 1-month (a), 6-month (b), and 12-month (c) follow-up after treatment with dexamethasone implant.

The analysis on 1-month BCVA change after DEX included one study from the ERM group and 2 studies from the RRD group. This analysis showed a significant visual improvement in the overall population (MD = −0.30, 95% CI = −0.39, −0.21; *p* < 0.001; Figure 3) and in both the ERM and RRD groups (ERM group, MD = −0.31, 95% CI = −0.42, −0.20, *p* < 0.001; RRD group, MD = −0.28, 95% CI = −0.45, −0.10, *p* < 0.001). No significant heterogeneity was found (overall, *I*^2^ = 0.01%, *p*=0.74; RRD group, *I*^2^ = 25.9%, *p*=0.25).

The analysis on 6-month BCVA change after DEX included 3 studies from the ERM group and 1 study from the RRD group. This analysis showed a significant visual improvement in the overall population (MD = −0.34, 95% CI = −0.43, −0.24; *p* < 0.001; Figure 3) and in both the ERM and RRD groups (ERM group, MD = -0.35, 95% CI = −0.48, −0.22, *p* < 0.001; RRD group, MD = −0.30, 95% CI = −0.48, −0.12, *p* < 0.001). Overall, no significant heterogeneity was found (*I*^2^ = 47.5%, *p*=0.13), but this was borderline nonsignificant when considering studies in the ERM group (*I*^2^ = 67.9%, *p*=0.06).

The analysis on 12-month BCVA change after DEX included a total of 4 studies, two from each group. This analysis showed a significant visual improvement in the overall population (MD = −0.26, 95% CI = −0.39, −0.13; *p* < 0.001; Figure 3) and in the ERM group (MD = −0.29, 95% CI = −0.42, −0.17, *p* < 0.001). BCVA change was nonsignificant in the RRD group (MD = −0.21, 95% CI = −0.52, 0.10, *p*=0.328). Significant heterogeneity was found overall (*I*^2^ = 70.5%, *p*=0.03). And in the RRD group (*I*^2^ = 81.4%, *p*=0.02), there was no significant heterogeneity in the ERM group (*I*^2^ = 64.4%, *p*=0.09).

No analysis was performed at a 3-month follow-up due to a lack of data.

### 3.5. Macular Thickness Outcome

**Figure 4:**
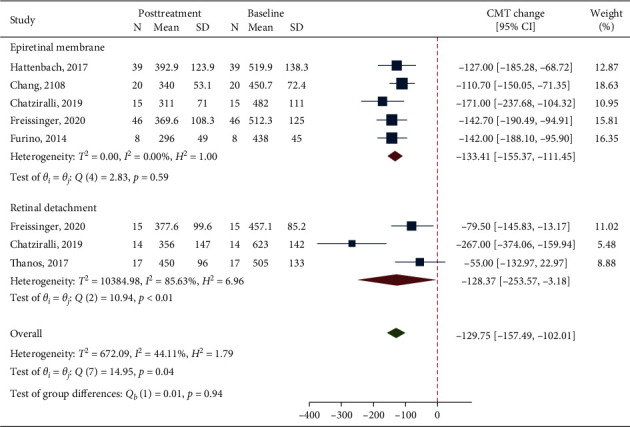
A forest plot showing mean change in central macular thickness considering the last available follow-up after treatment with dexamethasone implant.

Data from 5 studies and 3 studies were pooled together for CMT analysis in the ERM and RRD groups, respectively. The analysis on CMT change between baseline and last available follow-up after DEX showed a significant thickness reduction in the overall population, irrespective of the indication for vitrectomy (MD = −129.75, 95% CI = −157.49, −102.01; *p* < 0.001; Figure 4), with moderate but significant heterogeneity (*I*^2^ = 44.1%; *p*=0.04). In the ERM group, mean CMT significantly decreased following dexamethasone implant (MD = −133.41, 95% CI = −155.37, −111.45; *p* < 0.001), and no heterogeneity was found across studies (*I*^2^ = 0%; *p*=0.59). In the RRD group, the change between baseline and postdexamethasone CMT was borderline significant given a wide CI (MD = −128.37, 95% CI = −253.57, −3.18; *p*=0.040). In this group, significant heterogeneity was shown (*I*^2^ = 85.6%, *p* < 0.01). No difference in CMT improvement was found between the two groups (*p*=0.94).

**Figure 5:**
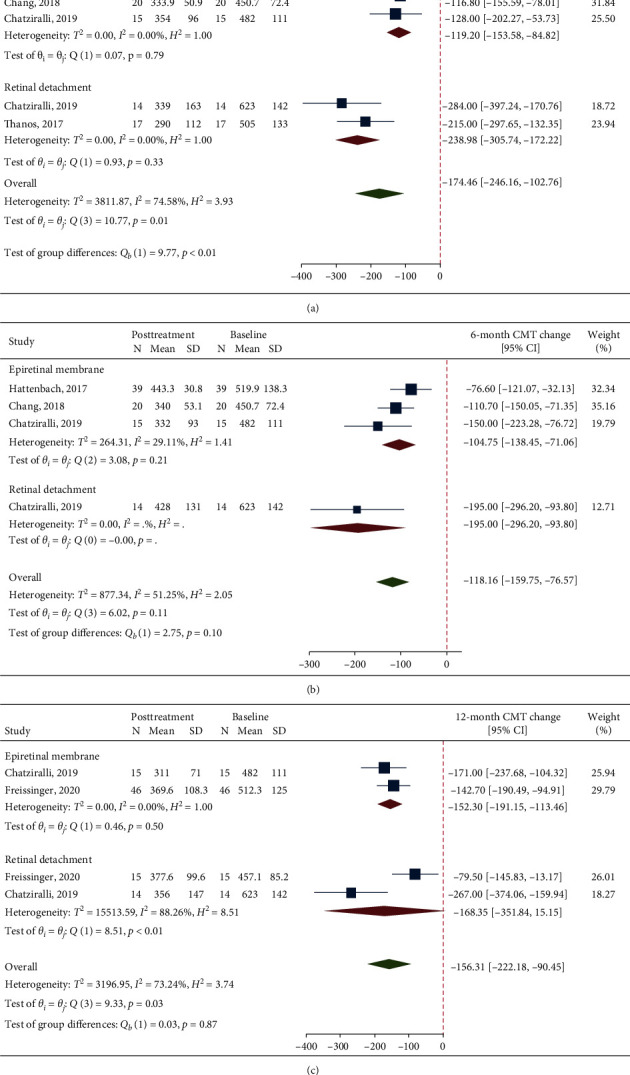
A forest plot showing mean change in central macular thickness at 1-month (a), 6-month (b), and 12-month (c) follow-up after treatment with dexamethasone implant.

The analysis on 1-month CMT change after DEX included 4 studies, two from each group. This analysis showed a significant CMT reduction in the overall population (MD = −174.76, 95% CI = −246.16, −102.76; *p* < 0.001; Figure 5) and in both the ERM and RRD groups (ERM group, MD = −119.20, 95% CI = −153.58, −84.82, *p* < 0.001; RRD group, MD = −238.98, 95% CI = −305.74, −172.22, *p* < 0.001). Significant heterogeneity was found when pooling all 4 studies together (*I*^2^ = 74.6%, *p*=0.01), while this was absent within both the ERM and RRD groups.

The analysis on 6-month CMT change after DEX included 3 studies from the ERM group and 1 study from the RRD group. This analysis showed a significant CMT reduction in the overall population (MD = −118.16, 95% CI = −159.75, −76.57; *p* < 0.001; Figure 5) and in both the ERM and RRD groups (ERM group, MD = −104.75, 95% CI = −138.45, −71.06, *p* < 0.001; RRD group, MD = −195.00, 95% CI = −296.20, −93.80, *p* < 0.001). No significant heterogeneity was found (overall, *I*^2^ = 51.3%, *p*=0.11; ERM group, *I*^2^ = 29.1%, *p*=0.21).

The analysis on 12-month CMT change after DEX included a total of 4 studies, two from each group. This analysis showed a significant CMT reduction in the overall population (MD = −156.31, 95% CI = −222.18, −90.45; *p* < 0.001; Figure 5) and in the ERM group (MD = −152.30, 95% CI = −191.15, −113.46, *p* < 0.001). CMT change was nonsignificant in the RRD group (MD = −168.35, 95% CI = −351.84, 15.15, *p*=0.456). Significant heterogeneity was found overall (*I*^2^ = 73.2%, *p*=0.03) and in the RRD group (*I*^2^ = 88.3%, *p* < 0.01); there was no significant heterogeneity in the ERM group (*I*^2^ = 0%, *p*=0.50).

No analysis was performed at a 3-month follow-up due to a lack of data.

## 4. Discussion

The present meta-analysis showed favorable visual and anatomical outcomes following the use of dexamethasone implant for macular oedema secondary to ERM and RRD vitrectomy.

Postvitrectomy macular oedema is a sight-threatening condition which could affect visual recovery following a successful surgery. This complication has been reported in roughly 15% of cases following RRD vitrectomy [18], while its incidence ranges from 13% to 47% following ERM vitrectomy [2, 19].

The causative mechanisms of postvitrectomy macular oedema have not been completely understood yet. It seems that inflammation plays a key role in this process. Indeed, macular oedema following RRD vitrectomy has been associated with the presence of proliferative vitreoretinopathy (PVR) and with longstanding RRD, which, in both cases, are likely to be linked with an inflammatory status [7]. Furthermore, macula-off RRD has been associated with a higher rate of postvitrectomy macular oedema [7]. It would be interesting to assess whether internal limiting membrane peeling could reduce its onset as this maneuver proved to reduce the risk of both postoperative ERM and RRD recurrence [20]. In case of ERM vitrectomy, macular distortion due to the contractile membrane has been assumed to trigger the inflammatory condition [21].

In this context, the use of the intravitreal 0.7 mg dexamethasone implant has been investigated. DEX is characterized by a potent anti-inflammatory activity and a good safety profile [22]. The implant is licensed in Europe for the treatment of posterior segment inflammation secondary to noninfectious uveitis, macular oedema due to retinal vein occlusion, and diabetic macular oedema (DMO) [23]. Additionally, DEX use has been also reported in other conditions with an inflammatory background, such as pseudophakic cystoid macular oedema [5], inflammation secondary to RRD repair surgery [24], and DMO worsening due to cataract surgery [25].

A remarkable advantage of the slow release implant is its efficacy in vitrectomized eyes, which are less suitable to intravitreal antivascular endothelial growth factor (anti-VEGF) therapy because of a faster washout [26].

Our findings showed both visual and anatomical improvements following DEX treatment for macular oedema secondary to vitrectomy for ERM and RRD.

When considering the last available follow-up, our analyses revealed a significant visual gain following DEX administration in both the ERM and the RRD groups, and in the overall population as well. Similarly, a significant reduction in macular thickness was shown in the overall population. Such an anatomical improvement was evident in the ERM group, while it was borderline significant in the RRD group due to a wide confidence interval.

When considering the different follow-ups, significant visual and anatomical improvements were demonstrated in both groups at 1 and 6 months. At 12 months, significant visual and anatomical improvements were shown in the ERM group, while these were nonsignificant in the RRD group.

While pseudophakic cystoid macular oedema has been widely studied and its spontaneous resolution has been reported up to 90% of cases [27, 28], less evidence is available on the natural history of postvitrectomy macular oedema. Both Chatziralli et al. [6] and Chang et al. [11], the two controlled studies included in this review, showed an unchanged, greater than 400 *µ*m central macular thickness in the untreated control group at the end of a 12- and 6-month follow-up, respectively. Additionally, Chatziralli et al. [6] reported a spontaneous resolution of macular oedema in only 33% of control cases. This might suggest that postvitrectomy macular oedema could be less prone to spontaneous resolution compared with pseudophakic cystoid macular oedema. However, evidence from only two small cohorts of control patients seems too limited to draw any conclusion. Both the two controlled studies included in this review reported on macular oedema secondary to ERM vitrectomy [6, 11], and even less is known on the natural history of macular oedema secondary to RRD vitrectomy. Further studies are warranted to better explore this issue.

Importantly, most of the included studies reported on persistent postvitrectomy macular oedema, which proved unresponsive to topical nonsteroidal anti-inflammatory drugs (NSAIDs) and/or periocular or intravitreal triamcinolone acetonide [8–12]. Only two studies included naïve patients [6, 13]. Chronic postsurgical macular oedema is unlikely to resolve spontaneously and its treatment might prove challenging [5]. The fact that DEX provided both a functional and anatomical improvement in vitrectomized eyes with, in most cases, persistent CMO is worth noting, in particular taking into account that a low number of implants (from 1 to 1.7) was administered over a follow-up ranging from 3 to 12 months.

In this systematic review, we collected DEX-related adverse events, as well. Of note, no case of endophthalmitis was reported by the included studies. In general, the main adverse events related to dexamethasone implant are IOP rise and cataract [23]. Most eyes of the included studies were pseudophakic at the time of DEX implant. This could be explained by the fact that these eyes had undergone a previous vitrectomy and cataract surgery could have been performed at that time or before.

With regard to IOP rise, the included studies reported this event from 0% to 20% of cases [8, 12]. It is important to point out that a higher rate of ocular hypertension was found in vitrectomized eyes compared to nonvitrectomized ones [29]. Theoretically, the implant could get worse in this condition. However, the included studies showed that all cases were amenable with IOP lowering drops, and in no case, surgery was required.

The following limitations characterized the present study. First, significant heterogeneity was found for BCVA analysis in both groups and for CMT analysis in the RRD group. The presence of high heterogeneity limits the quality of the evidence we provide. The reason for it could be related to the retrospective nature of most of the included studies and to the fact that different eligibility criteria and clinical variables could have been considered by the included studies. Additionally, no analysis was conducted on potential adverse events, such as IOP rise and cataract, due to the limited number of cases reported. Finally, a relatively small number of studies was included. However, a meta-analysis is featured by a greater power and accurate confidence interval compared with an individual study [30, 31].

In conclusion, the use of intravitreal dexamethasone implant for macular oedema following vitrectomy for ERM and RRD allowed improving both visual and anatomical outcomes. The implant represents a valid therapeutic option for this sight-threatening condition.

## Figures and Tables

**Table 1:** Characteristics of the included studies.

Type of surgery	Study	Number of eyes	Mean age	Follow-up month	Mean number of implants at end of follow-up	Lens status before implant
*ERM*	Furino et al. [12]	8	74	6, 75	1	8 pseudophakic
	Hattenbach et al. [8]	39	71, 5	4, 5	1, 59	1 phakic, 38 pseudophakic
	Chang et al. [11]	20	63, 9	6	1	20 pseudophakic
	Chatziralli et al. [6]	15	68, 2	12	1, 2	4 phakic, 11 pseudophakic
	Freissinger et al. [9]	46	66, 2	12	1, 67	24 phakic, 22 pseudophakic

*RRD*	Freissinger et al. [9]	15	60, 5	12	1, 3	3 phakic, 12 pseudophakic
	Chatziralli et al. [11]	14	56, 3	12	1, 4	6 phakic, 8 pseudophakic
	Thanos et al. [10]	17	67	3	1	17 pseudophakic

ERM: epiretinal membrane; RRD: rhegmatogenous retinal detachment.

## Data Availability

The data used to support the findings of this study are available in the Supplementary Information file.

## Supplementary Materials

Supplementary Materials Table S1: PRISMA 2009 checklist. Table S2: risk of bias of included studies according to the Methodological Item for Non-Randomized Studies (MINORS). Figure S1: funnel plot for best-corrected visual acuity change. Figure S2: funnel plot for central macular thickness change.
